# Smoking Cessation by Phone Counselling in a Lung Cancer Screening Program: A Retrospective Comparative Cohort Study

**DOI:** 10.1155/2022/5446751

**Published:** 2022-04-21

**Authors:** Ankita Ghatak, Sean Gilman, Siobhan Carney, Anne V Gonzalez, Andrea Benedetti, Nicole Ezer

**Affiliations:** ^1^Division of Experimental Medicine, Department of Medicine, McGill University, Montreal, Quebec, Canada; ^2^Department of Medicine, Division of Respiratory Medicine, McGill University Health Centre, Montreal, Quebec, Canada; ^3^Research Institute of the McGill University Health Centre, Montreal, Quebec, Canada; ^4^Center for Outcomes Research and Evaluation, McGill University Health Centre, Montreal, Quebec, Canada; ^5^Department of Epidemiology, Biostatistics & Occupational Health, McGill University, Montreal, Quebec, Canada

## Abstract

**Introduction:**

Smoking cessation integration within lung cancer screening programs is challenging. Currently, phone counselling is available across Canada for individuals referred by healthcare workers and by self-referral. We compared quit rates after phone counselling interventions between participants who self-refer, those referred by healthcare workers, and those referred by a lung cancer screening program.

**Methods:**

This is a retrospective cohort study of participants referred to provincial smoking cessation quit line in contemporaneous cohorts: self-referred participants, healthcare worker referred, and those referred by a lung cancer screening program if they were still actively smoking at the time of first contact. Baseline, covariates (sociodemographic information, smoking history, and history of mental health disorder) and quit intentions (stage of change, readiness for change, previous use of quit programs, and previous quit attempts) were compared among the three cohorts. Our primary outcome was defined as self-reported 30-day abstinence rates at 6 months. Multivariable logistic regression was used to identify whether group assignment was associated with higher quit rates.

**Results:**

Participants referred by a lung cancer screening program had low quit rates (12%, 95% CI: 5–19) at six months despite the use of phone counselling. Compared to patients who were self-referred to the smoking cessation phone helpline, individuals referred by a lung cancer screening program were much less likely to quit (adjusted OR 0.37; 95% CI: 0.17–0.8), whereas those referred by healthcare workers were twice as likely to quit (adjusted OR 2.16 (1.3–3.58)) even after adjustment for differences in smoking intensity and quit intentions.

**Conclusions:**

Phone counselling alone has very limited benefit in a lung cancer screening program. Participants differ significantly from those who are otherwise referred by healthcare workers. This study underlines the importance of a dedicated and personalized tobacco treatment program within every lung cancer screening program. The program should incorporate best practices and encourage treatment regardless of readiness to quit.

## 1. Introduction

Across Canada, multiple provinces are implementing lung cancer screening following results from multiple large trials showing a mortality benefit of low-dose CT (LDCT) screening [1–4] and cost-effectiveness in Canada [5]. Given the significant mortality benefit of smoking cessation in combination with lung cancer screening [6], as well as the high proportion of active smokers in screening programs, there is interest using engagement in screening as a teachable moment to promote smoking cessation. In a secondary analysis of the National Lung Screening Trial, the 7-year smoking abstinence in the control arm (i.e., who underwent chest X-ray) was equivalent to a 20% reduction in lung cancer-specific mortality [6]. The authors note that this reduction is equivalent to the mortality benefit of three annual CT screening rounds. Combined abstinence and CT screening were associated with an almost two-fold increase in benefit, resulting in a 38% reduction in lung cancer death, HR: 0.62 (95% CI: 0.51–0.76). Smoking cessation provides an additional 10% mortality benefit when combined with LDCT screening [1, 6].

Participation in a lung cancer screening program without a smoking cessation intervention has been shown not to increase quit rates [3]. In the Cancer Care Ontario pilot lung cancer screening program, in-person counselling is offered to participants [7]. However, in the context of reduced healthcare resources, since the onset of the COVID-19 pandemic, many programs may only be able to integrate smoking cessation services into existing phone counselling programs because of lack of manpower. Studies assessing phone counselling in patients screened for lung cancer as part of clinical trials are limited [8–10]. Little information is available on the comparative effectiveness of programs between participants referred by healthcare workers (nurses, community pharmacists, and physicians), participants who are self-referred, and those referred by a lung cancer screening program. There is a need for evidence-based data for provincial lung cancer screening programs as they deploy resources to increase smoking cessation rates among participants. Additionally, understanding how referred participants differ from those traditionally referred to smoking quit lines will allow for interventions to be tailored addressing these differences.

We performed a retrospective cohort study to assess the effectiveness of telephone counselling for smoking cessation in lung cancer screening eligible participants from the McGill Lung Cancer Screening Pilot program to participants referred by healthcare workers and those who self-refer to Quebec's smoking cessation helpline (Ligne J'arrete).

## 2. Materials and Methods

Participants were identified as self-referred, those referred by healthcare workers in the context of usual clinical care by nurses, pharmacists, or their family doctors, and those referred by the McGill Lung Cancer Screening Pilot program between 2019 and 2020. Participants were matched by date and referral status 2 : 2 : 1 ratio. Participants who called the lung cancer screening program and identified as active smokers at time of first contact with the program were referred to the smoking cessation quit line. Participants who engaged with the lung cancer screening program were those who had at a minimum one phone counselling intervention with the quit line, and we excluded participants who had quit smoking by the time they were contacted by the quit program. The lung cancer screening program followed Quebec's Institut national d'excellence en santé et services sociaux recommendation to determine lung cancer screening eligibility with a 6-year lung cancer risk greater than or equal to 2% using the prostate, lung, colorectal, and ovarian cancer (PLCO) m2012 risk prediction model [5]. Participants who were not eligible for screening based on PLCO and/or age but initiated contact with the program and identified as active smokers were referred to the quit line and were included in our analysis.

Baseline sociodemographic information such as age, sex, and highest educational attainment (less than high school, some training after high school, high school graduate, college graduate, and postgraduate) were collected in all participants. The history of mental health disorder (anxiety, bipolar, clinical depression, seasonal depression, pathological gambling, schizophrenia, eating disorder, border personality disorder, and drug or alcohol use) was collected by the smoking cessation phone line by self-report.

To quantify smoking, we collected time to first cigarette (within the first 5 minutes after waking up, between 6 and 30 minutes after waking up, between 31 and 60 minutes after waking up, and more than 60 minutes), number of cigarettes used per day (at baseline), and heaviness of smoking index. Heaviness of smoking index uses a 6-point scale and combines data on baseline cigarette use per day and time to first cigarette. It was used to compare nicotine dependance in the three groups [11]. Participants' stage of change was categorized using the transtheoretical model of behavioral stages of change to categorize them as precontemplation (no thoughts of quitting), contemplation (thinking about quitting), preparation (planning to quit in the next 30 days), action (quitting successfully for up to six months), and maintenance (no smoking for more than six months) [12]. Previous quit attempts, previous use of pharmacological intervention (nicotine patches and gum), and previous use of quit lines were defined as “yes/no.” Readiness for change, both importance and confidence in quitting, were measured on a 10-point scale, with 1 being very low and 10 being very high.

The primary outcome was self-reported 30-day abstinence rates at 6 months after first contact with the phone quit line. Baseline sociodemographic information, history of mental health disorders and smoking data, and heaviness of smoking index were compared between the three groups using a chi-squared test, one-way ANOVA, or Kruskal–Wallis test where appropriate. Quit intentions were compared in the three groups using a chi-squared test.

Missing data were imputed using multiple imputations by chained equation (MICE) using 250 iterations and pooled 50 imputed datasets to get the final dataset [13]. A multivariate logistic regression was developed to determine the impact of group allocation using the following variables of interest: age, gender (male, female), education (collapsed into less than high school, more than high school), time to first cigarette (collapsed into within 5 minutes after waking up, more than 5 minutes after waking up), cigarette use per day (at baseline), and group (self-referred, healthcare worker referred, McGill Lung Cancer Screening Pilot program referred).

All data were cleaned and analyzed using Python [14] and R [13]. The study was approved by the Research Ethics Board of the McGill University Health Center.

## 3. Results and Discussion

### 3.1. Results

A total of 417 active smokers were included in the study, 176 (42%) were self-referred arm, 165 (40%) were referred by health care workers (family doctors, nurses, pharmacists), and 76 (18%) were referred by the lung cancer screening program. Three individuals were excluded from the study as they had quit smoking after contacting the lung cancer screening program and prior to being contacted by the smoking cessation quit line. As expected, mean age was highest in the lung cancer screening referred group (63 years, standard deviation (SD) 6) and was lower in the self-referred (53 years, SD 15) and healthcare worker referred (49 years, SD 13) groups (*p* < 0.001). The lung cancer screening referred group had lower educational attainment, with the majority of participants (26.3%) having a less than a high school education (*p* < 0.001), and those referred by healthcare workers had higher educational attainment (32.1% college graduates and 20.6% postgraduates).

There were differences in nicotine dependence between the three groups. Overall, the majority of participants smoked within 5 minutes of waking up (48.7%) or within 6 and 30 minutes of waking up (24.2%). However, this trend was the same in the three groups: self-referred (55.1% within 5 minutes of waking up, and 19.3% within 6 and 30 minutes of waking up), healthcare worker referred (46.7% and 23.6%), and lung cancer screening referred (38.2% and 36.8%) (*p* < 0.001). This analysis was limited by the small number individuals in each cell. The mean number of cigarettes used per day was highest amongst those referred by the lung cancer screening program (median 20 per day; IQR 13–25), followed by the self-referred group (median 18 per day; IQR 10–25) and the healthcare worker referred group (median 16 per day, IQR 10–25), although the difference was not statistically significant between the three groups (*p* > 0.05). Additionally, participants had on average moderate nicotine dependance defined using the heaviness of index. Despite the higher number of cigarettes used per day in the lung cancer screening group, the heaviness of smoking index was similar in the three groups (*p* > 0.05) (Table 1).

Stage of change was significantly different in the three groups (*p* < 0.001) (Table 2). Among the participants referred by the lung cancer screening program, the majority were in the first three stages of change—precontemplation (15.8%), contemplation (27.6%), and preparation (43.4%). By comparison, very few self-referred and healthcare worker referred participants were in the precontemplation (5.7% and 4.8%, respectively) or contemplation stages (8.5% and 10.3%, respectively). Among participants referred by healthcare workers, most were in the action stage (48.5%) compared to only 9.2% among lung cancer screening referred participants. Participants' quit histories different significantly between groups. Considerably, more lung cancer screening referred participants had had previous quit attempts (36.8%) as compared to self-referred and healthcare worker referred participants (11.9% and 10.9%, respectively) (*p* < 0.001). A larger proportion of lung cancer screening referred participants reported previous use of pharmacological therapy (30.3%) as compared to self-referred and healthcare worker referred participants (24.4% and 12.7%) (*p* < 0.01). Across all groups, the majority of participants reported previous use of quit lines, with the highest being amongst the healthcare worker referred group (94.5%), followed by the lung cancer screening referred group (92.1%) and the self-referred group (84.1%) (*p* < 0.01). Notably, a higher proportion of lung cancer screening referred participants reported mental health disorders (47.4%) as compared to both self-referred and healthcare worker referred participants (39.2% and 28.5%, respectively) (*p* < 0.05). Both readinesses for change measures differed significantly between the three groups. Approximately half of all lung cancer screening referred participants rated their readiness for change, importance in quitting as 5 (19.7%) or 6 (30.3%)—the lowest scores reported in the study—as compared to only a small percentage of self-referred (7.4% and 9.7%) and healthcare worker referred participants (2.4% and 7.9%). As such, a significantly lower proportion of lung cancer screening referred participants rated their importance in quitting as 10 (23.7%) compared to self-referred (57.4%) and healthcare worker referred participants (63%) (*p* < 0.001). Similarly, very few lung cancer screening referred participants rated their readiness for change, confidence in quitting, as 10 (2.6%) compared to self-referred (7.4%) and healthcare worker referred participants (*p* < 0.001) (Table 2).

Overall, 30-day abstinence at 6 months was 30% among all participants (Table 3). Six-month quit rates were the lowest amongst participants referred by the lung cancer screening program (12%, 95% CI: 5–19), and highest amongst participants referred by healthcare workers (42%, CI: 35–50) (*p* < 0.001). After adjustment for sex, age, education (less than high school or high school or more), baseline cigarette use per day, and time to first cigarette (less than or more than 5 minutes from waking up), participants who were referred by healthcare workers were almost twice as likely to quit than those who were self-referred (adjusted OR: 2.12, 95% CI: 1.29–3.51); whereas, those participants who were referred by the lung cancer screening program were significantly less likely to quit, even after adjustment (adjusted OR: 0.34, 95% CI: 0.15–0.76) (Table 4).

## 4. Discussion

Combining smoking cessation with lung cancer screening by low-dose CTs has been shown to be associated with a 38% reduction in death from lung cancer [6]. Although it is evident that smoking cessation should be incorporated into screening programs [15], there is limited evidence on how best to integrate these services. Across studies of participants screened for lung cancer, quit rates with no smoking cessation intervention range 7–23% [16]. Our 6-month quit rate of 12% (95% CI: 5–19) among individuals screened for lung cancer is comparable to similar studies with no smoking cessation intervention [4]. Our quit rates are unchanged even after adjustment for age, smoking intensity, and education. Our results show that phone counselling as a smoking cessation intervention did not increase these quit rates at 6 months among participants referred by a lung cancer screening program. Whether smokers advanced through states of change from a precontemplation state of change to a contemplation state is still unknown. This lack of effect on quit rates is of significant concern given the significant cost and resources needed to absorb the added volume to smoking cessation referrals to quit lines if they systematically will be targeting participants in screening programs. Additionally, data like these are important to differentiate the effect of the quit lines themselves from the effect of engagement with a screening program which is likely a teachable moment for participants.

Smoking cessation quit lines have two different roles in smokers and ex-smokers. In smokers, their role is to encourage smoking cessation. In ex-smokers who are in the action and maintenance phases of smoking cessation, their role is to avoid smoking relapses. In smokers, the prevalent view is that participants referred by a lung cancer screening program to a phone quit line are similar to participants referred by healthcare workers. However, our results demonstrate this is definitely not the case. Participants demonstrate key differences. Participants referred by the lung cancer screening program have a higher age, lower educational attainment, and a higher number of previous failed smoking cessation attempts. Additionally, they show differences in quit intentions and readiness for change, despite similar use of quit programs in the past. Most notably, a referral by a healthcare worker outside of a lung cancer screening program is likely a sign that an individual is in the “action” stage of change, whereas our participants were more likely to be in precontemplation or contemplation stages of change. These differences are important for screening programs in the process of implementing smoking cessation interventions to tailor interventions to focus on state of change and educational attainment in order to improve success rates.

Some studies suggest that findings after lung cancer screening using low-dose CT is performed would help tailor the intervention to encourage cessation using personalized results of the screening study. However, a recently published Canadian randomized control trial of a telephone-based smoking cessation intervention demonstrated incorporating lung cancer screening results did not result in increased 12-month cessation rates versus written information alone in unselected smokers undergoing lung cancer screening [9]. To optimize cessation interventions in this population, behavioral counselling combined with pharmacotherapy are more promising than telephone counselling alone. Cessation rates have been demonstrated to be up to 57% with these strategies in the first six months in clinical trials [17–20]. Beneficial effects decline after a year and participants increasingly relapse with passage of time, and follow-up sessions might be required to maintain treatment effects [18]. Internet-based interventions such as computer-tailored cessation advice or a list of internet resources has not shown to be beneficial over standard written information material [21, 22].

Our study is limited by our short follow-up time of 6 months and the fact that smoking cessation was not confirmed biochemically. Nevertheless, verbal assessment alone is likely to overestimate the effectiveness of the intervention. Additionally, we used multiple imputations to deal with missing data and under the assumption that data were missing at random. Notably, a complete case analysis showed similar numbers also before and after adjustment, with the lung cancer screening referred group still being significantly less likely to quit, especially compared to the healthcare worker referred group, thus supporting our results.

## 5. Conclusions

These findings, along with those from another Canadian randomized clinical trial of smoking cessation integration into a lung cancer screening trial [9], have important implications for lung cancer screening programs across Canada. They suggest options other than phone counselling, such as multimodality interventions with in-person motivational interviewing and pharmacotherapy, are more likely to demonstrate clinical effectiveness for lung cancer screening participants.

## Figures and Tables

**Table 1:** Baseline demographics.

	Self-referred *n* = 176	Healthcare worker referred *n* = 165	Lung cancer screening referred *n* = 76	Total *n* = 417	*P* value
Age, mean (SD)	53 (15)	49 (13)	63 (6)	53 (14)	<0.001
Age, median (Q1, Q3)	57 (39, 65)	48 (39, 59)	63 (59, 67)	57 (40, 64)	
Sex, *n* (%)					
F	90 (51)	108 (65.5)	33 (43.4)	231 (55.4)	0.002
M	86 (48.9)	57 (34.5)	43 (56.6)	186 (44.6)	
Education, *n* (%)					
Less than high school	55 (31.2)	15 (9.1)	20 (26.3)	90 (21.6)	<0.001
Some training after high school	17 (9.7)	24 (14.5)	17 (22.4)	58 (13.9)	
High school graduate	44 (25.0)	39 (23.6)	12 (15.8)	95 (22.8)	
College graduate	37 (21.0)	53 (32.1)	9 (11.8)	99 (23.7)	
Postgraduate	23 (13.1)	34 (20.6)	18 (23.7)	75 (18.0)	
Baseline cigarette use per day, mean (SD)	18 (10)	18 (11)	20 (9)	19 (11)	0.471
Baseline cigarette use per day, median (Q1, Q3)	18 (10, 25)	16 (10, 25)	20 (13, 25)	18 (10, 25)	0.283
Time to first cigarette, *n* (%)					
Within the first 5 minutes after waking up	97 (55.1)	77 (46.7)	29 (38.2)	203 (48.7)	<0.001
Between 6 and 30 minutes after waking up	34 (19.3)	39 (23.6)	28 (36.8)	101 (24.2)	
Between 31 and 60 minutes after waking up	14 (8.0)	14 (8.5)	15 (19.7)	43 (10.3)	
More than 60 minutes after waking up	31 (17.6)	35 (21.2)	4 (5.3)	70 (16.8)	
Heaviness of smoking index, mean (SD)	3 (2)	3 (2)	3 (1)	3 (2)	0.415
Heaviness of smoking index, median (Q1, Q3)	4 (2, 5)	3 (2, 5)	3 (3, 4)	3 (2, 5)	0.652

**Table 2:** Quit intentions.

	Self-referred *n* = 176	Healthcare worker referred *n* = 165	Lung cancer screening referred *n* = 76	Overall *n* = 417	*P* value
Stage of change, *n* (%)					
Precontemplation	10 (5.7)	8 (4.8)	12 (15.8)	30 (7.2)	<0.001
Contemplation	15 (8.5)	17 (10.3)	21 (27.6)	53 (12.7)	
Preparation	100 (56.8)	59 (35.8)	33 (43.4)	192 (46.0)	
Action	44 (25.0)	80 (48.5)	7 (9.2)	131 (31.4)	
Maintenance	7 (4.0)	1 (0.6)	3 (3.9)	11 (2.6)	
Previous quit attempts, *n* (%)					
No	21 (11.9)	18 (10.9)	28 (36.8)	67 (16.1)	<0.001
Yes	155 (88.1)	147 (89.1)	48 (63.2)	350 (83.9)	
Previous use of pharmacological therapy, *n* (%)					
No	43 (24.4)	21 (12.7)	23 (30.3)	87 (20.9)	0.002
Yes	133 (75.6)	144 (87.3)	53 (69.7)	330 (79.1)	
Previous use of quit lines, *n* (%)					
No	28 (15.9)	9 (5.5)	6 (7.9)	43 (10.3)	0.005
Yes	148 (84.1)	156 (94.5)	70 (92.1)	374 (89.7)	
Mental health disorders, *n* (%)					
No	107 (60.8)	118 (71.5)	40 (52.6)	265 (63.5)	0.011
Yes	69 (39.2)	47 (28.5)	36 (47.4)	152 (36.5)	
Readiness for change—importance in quitting, *n* (%)					
10	101 (57.4)	104 (63.0)	18 (23.7)	223 (53.5)	<0.001
9	7 (4.0)	20 (12.1)	7 (9.2)	34 (8.2)	
8	27 (15.3)	17 (10.3)	4 (5.3)	48 (11.5)	
7	11 (6.2)	7 (4.2)	9 (11.8)	27 (6.5)	
6	17 (9.7)	13 (7.9)	23 (30.3)	53 (12.7)	
5	13 (7.4)	4 (2.4)	15 (19.7)	32 (7.7)	
Readiness for change—confidence in quitting, *n* (%)					
10	13 (7.4)	27 (16.4)	2 (2.6)	42 (10.1)	<0.001
9	16 (9.1)	13 (7.9)	3 (3.9)	32 (7.7)	
8	46 (26.1)	49 (29.7)	13 (17.1)	108 (25.9)	
7	27 (15.3)	23 (13.9)	11 (14.5)	61 (14.6)	
6	10 (5.7)	19 (11.5)	1 (1.3)	30 (7.2)	
5	15 (8.5)	12 (7.3)	3 (3.9)	30 (7.2)	
4	3 (1.7)	3 (1.8)	8 (10.5)	14 (3.4)	
3	38 (21.6)	16 (9.7)	24 (31.6)	78 (18.7)	
2	8 (4.5)	3 (1.8)	11 (14.5)	22 (5.3)	

**Table 3:** Smoking status of participants at 6 months.

	Self-referred *n* = 176	Healthcare worker referred *n* = 165	Lung cancer screening referred *n* = 76	Overall *n* = 417	*P* value
Smoking status, *n* (%, 95% CI)					
Smoker	129 (73%, 95% CI: 67–80)	95 (58%, 95% CI: 50–65)	67 (88%, 95% CI: 81–95)	291 (70%, 95% CI: 65–74)	<0.001
Quitter	47 (27%, 95% CI: 20–33)	70 (42%, 95% CI: 35–50)	9 (12%, 95% CI: 5–19)	126 (30%, 95% CI: 26–35)	

Quitter is defined as self-reported 30-day abstinence rates at 6 months.

**Table 4:** Logistic regression with imputed values.

Group	Unadjusted	Adjusted^*∗*^
Healthcare worker referred	2.02 (1.29–3.20)	2.12 (1.29–3.51)
Lung cancer screening referred	0.37 (0.16–0.77)	0.34 (0.15–0.76)

Reference group is control 1 (self-referred). ^*∗*^Adjusted for sex, education, age, time to first cigarette (categorical), and baseline cigarette use per day (continuous).

## Data Availability

The anonymized data are stored on the RedCap Database of the Research Institute of the McGill University Health Center and are available from the corresponding author upon request.

## References

[1] Aberle D. R., Adams A. M., Berg C. D. (2011). Reduced lung-cancer mortality with low-dose computed tomographic screening. *New England Journal of Medicine*.

[2] de Koning H. J., van der Aalst C. M., de Jong P. A. (2020). Reduced lung-cancer mortality with volume CT screening in a randomized trial. *New England Journal of Medicine*.

[3] Pedersen J. H., Tønnesen P., Ashraf H. (2016). Smoking cessation and lung cancer screening. *Annals of Translational Medicine*.

[4] Pastorino U., Silva M., Sestini S. (2019). Prolonged lung cancer screening reduced 10-year mortality in the MILD trial: new confirmation of lung cancer screening efficacy. *Annals of Oncology*.

[5] Tammemagi M. C., Schmidt H., Martel S. (2017). Participant selection for lung cancer screening by risk modelling (the pan-canadian early detection of lung cancer [pancan] study): a single-arm, prospective study. *Lancet Oncology*.

[6] Tanner N. T., Kanodra N. M., Gebregziabher M. (2016). The association between smoking abstinence and mortality in the national lung screening trial. *American Journal of Respiratory and Critical Care Medicine*.

[7] Evans W. K., Truscott R., Cameron E. (2019). Implementing smoking cessation within cancer treatment centres and potential economic impacts. *Translational Lung Cancer Research*.

[8] Cadham C. J., Jayasekera J. C., Advani S. M. (2019). Smoking cessation interventions for potential use in the lung cancer screening setting: a systematic review and meta-analysis. *Lung Cancer*.

[9] Tremblay A., Taghizadeh N., Huang J. (2019). A randomized controlled study of integrated smoking cessation in a lung cancer screening program. *Journal of Thoracic Oncology*.

[10] Cahill K., Lancaster T., Green N. (2010). Stage-based interventions for smoking cessation. *Cochrane Database of Systematic Reviews*.

[11] Heatherton T. F., Kozlowski L. T., Frecker R. C., Rickert W., Robinson J. (1989). Measuring the heaviness of smoking: using self-reported time to the first cigarette of the day and number of cigarettes smoked per day. *Addiction*.

[12] Prochaska J. O., DiClemente C. C. (1983). Stages and processes of self-change of smoking: toward an integrative model of change. *Journal of Consulting and Clinical Psychology*.

[13] Buuren S. v., Groothuis-Oudshoorn K. (2011). Mice: multivariate imputation by chained Equations in R. *Journal of Statistical Software*.

[14] Pollard T. J., Johnson A. E. W., Raffa J. D., Mark R. G. (2018). Tableone: an open source python package for producing summary statistics for research papers. *JAMIA Open*.

[15] Fucito L. M., Czabafy S., Hendricks P. S., Kotsen C., Richardson D., Toll B. A. (2016). Pairing smoking-cessation services with lung cancer screening: a clinical guideline from the association for the treatment of tobacco use and dependence and the society for research on nicotine and tobacco. *Cancer*.

[16] Moldovanu D., de Koning H. J., van der Aalst C. M. (2021). Lung cancer screening and smoking cessation efforts. *Translational Lung Cancer Research*.

[17] Pistelli F., Aquilini F., Falaschi F. (2020). Smoking cessation in the ITALUNG lung cancer screening: what does “teachable moment” mean?. *Nicotine & Tobacco Research*.

[18] Pozzi P., Munarini E., Bravi F. (2015). A combined smoking cessation intervention within a lung cancer screening trial: a pilot observational study. *Tumori Journal*.

[19] Lucchiari C., Masiero M., Mazzocco K. (2020). Benefits of e-cigarettes in smoking reduction and in pulmonary health among chronic smokers undergoing a lung cancer screening program at 6 months. *Addictive Behaviors*.

[20] Filippo L., Principe R., Cesario A. (2015). Smoking cessation intervention within the framework of a lung cancer screening program: preliminary results and clinical perspectives from the “Cosmos-II” trial. *Lung*.

[21] van der Aalst C. M., de Koning H. J., van den Bergh K. A. M., Willemsen M. C., van Klaveren R. J. (2012). The effectiveness of a computer-tailored smoking cessation intervention for participants in lung cancer screening: a randomised controlled trial. *Lung Cancer*.

[22] Clark M. M., Cox L. S., Jett J. R. (2004). Effectiveness of smoking cessation self-help materials in a lung cancer screening population. *Lung Cancer*.

